# Fulminant Hepatic Failure in the Course of an Outpatient Anesthetic Procedure: Sevoflurane among Other High-Risk Factors

**DOI:** 10.1155/2020/5124098

**Published:** 2020-06-29

**Authors:** Céline Cheron, Perrine Hoet, Nathalie Renard, Geoffroy Vanderweerden, Cristina Miscu, Mina Komuta, Pierre-François Laterre, Philippe Hantson

**Affiliations:** ^1^Department of Intensive Care, Université Catholique de Louvain, Cliniques St-Luc, 1200 Brussels, Belgium; ^2^Louvain Centre for Toxicology and Applied Pharmacology, Université Catholique de Louvain, 1200 Brussels, Belgium; ^3^Department of Pathology, Centre Hospitalier de Wallonie Picarde, Tournai, Belgium; ^4^Department of Anesthesiology, Centre Hospitalier de Wallonie Picarde, Tournai, Belgium; ^5^Department of Gastroenterology, Centre Hospitalier de Wallonie Picarde, Tournai, Belgium; ^6^Department of Pathology, Université Catholique de Louvain, Cliniques St-Luc, 1200 Brussels, Belgium

## Abstract

A 20-year-old man underwent an outpatient general anesthetic procedure with sevoflurane for the correction of a bilateral gynecomastia. The patient had been first exposed to sevoflurane two years before, without any complication. He presented an overweight with a body mass index (BMI) of 31.4 kg/m^2^ and had an episode of “binge” drinking a few days before anesthesia. He became icteric from postoperative day 9, and after the worsening of liver function tests, the liver biopsy revealed centrilobular necrosis. The patient became encephalopathic and required urgent liver transplantation on postoperative day 30. The possibility of a sevoflurane-related fulminant hepatic failure is discussed.

## 1. Introduction

With modern volatile anesthetics (VA), liver toxicity has become an uncommon complication. However, the risk of volatile anesthetic drug-induced liver injury (VA-DILI) has not totally disappeared, even with desflurane and sevoflurane [1]. This complication is likely a combination of toxicity and autoimmunity. Evolution into fulminant forms of hepatic failure is rare. The diagnosis requires the exclusion of other common causes of hepatic failure. We present a recent case with a high suspicion of severe sevoflurane-related DILI leading to urgent liver transplantation. We also discuss the possible mechanisms and risk factors.

## 2. Case Presentation

A 20-year-old man underwent elective general anesthesia with sevoflurane for the correction of a bilateral gynecomastia. He had no previous medical history, except for a correction of a nasal fracture that occurred two years ago, also under sevoflurane general anesthesia. He had no history of allergy or drug abuse. The preoperative liver tests were normal. His current body weight was 105 kg (183 cm height), corresponding to a body mass index (BMI) of 31.4 kg/m^2^. Five days before the current surgery, the patient admitted an episode of binge drinking at a party. The anesthetic and surgical procedures were uneventful, with no adverse hemodynamic event. The total duration of sevoflurane anesthesia was 93 minutes. The dose of sevoflurane was adjusted to keep the bispectral index (BIS) of the patient between 40 and 60. The mean alveolar concentration (MAC) was maintained between 0.8 and 1.2 during the whole procedure. During anesthesia or immediately after, the patient received dexamethasone 4 mg, midazolam 1.5 mg, ketamine 100 mg, clonidine 300 µg, lidocaine 100 mg, tracrium 50 mg, cefazolin 2 g, paracetamol 1 g, and ketorolac 30 mg. The postoperative course was not complicated, and the patient used only 2 g of paracetamol for pain relief postoperatively. Laboratory investigations at recovery were normal. Two days after surgery, when at home, he started to complain of pruritus. He became icteric on day 9 and was readmitted to the first hospital on day 15 with alteration of liver tests (bilirubin 12.7 mg/dl, ALT 966 IU/l, and alkaline phosphatase 259 IU/l), oliguria, prothrombin time 75% of normal activity, and no encephalopathy. Extensive laboratory (virology, serology, and autoimmunity) investigations were negative for the common etiologies of acute hepatitis, and a toxic origin was suspected. A liver biopsy was performed showing foci of centrilobular necrosis associated with a mixed lymphocytic and neutrophilic infiltrate and a mild degree of bile duct atrophy but no intrahepatic cholestasis. Due to the progression of cytolysis, he was then referred to a liver transplantation center on day 28. He presented several clinical and biological criteria attesting the severity of liver injury: encephalopathy grade 3-4, international normalized ratio (INR) > 7, bilirubin 27.4 mg/dl, factor V 14%, lactic acidosis (peak arterial lactate 9.4 mmol/l), and serum creatinine 117.9 *µ*mol/l. The peak of ALT was 3080 IU/l on day 22. There was no increase in eosinophil count. The patient was listed for urgent liver transplantation and was treated with plasma exchanges. A graft was available on day 30, and surgery was not complicated. The ultrastructural examination of the explanted liver showed an acute necrotizing hepatitis (“bridging necrosis”) without fibrosis, and residual parenchyma was around 30%; a severe inflammatory reaction was noted with a majority of lymphocytes. There was also discrete micro- and macrovesicular steatosis associated with ballooned hepatocytes and Mallory-Denk bodies, but no significant cholestasis.

The Roussel Uclaf Causality Assessment Method (RUCAM) score for DILI was 13. During the postoperative phase (day 10), the patient developed a severe neutropenia that was suspected toxic and drug-related as other etiologies were excluded.

## 3. Discussion

Among volatile halogenated anesthetics, halothane has been classically associated with various forms of liver injury in up to 24.4% of patients [1]. The most notable histological feature of halothane hepatitis is centrilobular necrosis, with a variable pattern of severity from patchy to massive necrosis. With the recent generation of volatile anesthetics (isoflurane, desflurane, and sevoflurane), the incidence of drug-induced liver injury (DILI) had markedly decreased [2]. A retrospective study has shown that liver injury induced by isoflurane, desflurane, or sevoflurane may not be as rare as was previously thought, although most cases are mild and not clinically significant. Three percent of 1556 patients had abnormal postoperative liver biochemistry potentially attributable to VA, 1% had significant VA drug‐induced liver injury, with an ALT rise between 200 and 900 U/L (5–22 × ULN), and no cases of fulminant hepatitis were seen [3].

Halothane in particular proved to be associated with a significant risk of fulminant hepatitis. There is no same concern regarding hepatotoxicity after anesthesia with sevoflurane, a “modern” VA, generally considered as much safer alternative with a low hepatotoxic potential. Nonetheless, sevoflurane anesthesia has been implicated in several cases of severe acute liver damage, with histological features similar as halothane-induced hepatitis [4]. The actual incidence and relationship of sevoflurane to these events cannot be established with certainty.

Halothane is metabolised by cytochrome P450 2E1 isoform (CYP2E1) to produce reactive trifluoroacetyl chloride (TFA), which binds to hepatic proteins forming neoantigens, responsible for the production of autoantibodies against liver tissue. Idiosyncratic drug-induced liver injury (IDILI) is suggested to be triggered by native hepatic proteins, such as CYP2E1, which has been covalently modified by TFA [5, 6]. Reactive metabolites can covalently bind proteins and exert direct toxicity, with alteration of function or location of the target protein, or form drug-protein adducts that might trigger immune-mediated reactions, but they can also cause no adverse effect or clinical impact, for instance, if only few proteins are modified [6]. In line with this, the levels of CYP2E1 (and ERp58) autoantibodies were increased in asymptomatic pediatric anesthesiologists compared to general anesthesiologists due to an increased environmental exposure to VA, suggesting that autoantibodies may not have a pathological role in VA-induced hepatitis [7]. Moreover, CYP2E1 autoantibodies are not specific of VA-DILI [8, 9]. Some findings suggest that people environmentally exposed to halothane, isoflurane, and desflurane develop CYP2E1-specific IgG1 autoantibodies. In contrast, patients with anesthetic-induced IDILI had significantly elevated levels of CYP2E1-specific IgG4 autoantibodies, while anesthetic-exposed healthy persons had significantly elevated levels of CYP2E1-specific IgG1 autoantibodies [10].

Unlike other anesthetic gases, sevoflurane does not have a reactive metabolite in its metabolic pathway, thus reducing the risk of hepatotoxicity [11]. TFA derivatives are required to create neoantigens. Experimental studies as well as human studies on patients, occupationally exposed subjects, and volunteers have shown that the biotransformation rate of sevoflurane is low. Between 95% and 98% sevoflurane is quickly eliminated via exhaled breath. Only a limited amount of absorbed sevoflurane undergoes a biotransformation, in which CYP2E1 plays a major role, to mainly inorganic and organic fluoride. Hexafluoroisopropanol (HFIP), the only organic fluoride metabolite identified to date, is rapidly conjugated with glucuronic acid in the liver and excreted in urine. There is no evidence that HFIP undergoes further defluorination or oxidative metabolism [12]. Covalent binding of TFA to the hepatic protein was not detectable in rats after exposure to sevoflurane or desflurane, whereas a significant amount was observed after halothane exposure [13]. To our knowledge, TFA has never been identified as a metabolite of sevoflurane: neither in animal studies nor in sevoflurane-anesthetized patients or occupationally exposed subjects, and covalent binding of sevoflurane to hepatic macromolecules is theoretically unlikely.

Our patient presented two conditions known to alter the hepatic metabolic balance drastically, obesity and a history of alcohol consumption, both conditions leading to the fatty liver, induction of CYP2E1 activity, and tissue hypoxia [14]. Accordingly, acute ethanol administration was shown to inhibit enflurane metabolism in rats. However, the interval of time between cessation of ethanol ingestion and administration of enflurane determined whether the biotransformation is inhibited, stimulated, or unchanged. Also, CYP2E1 induction was observed in mice in acute, binge, and chronic alcohol exposure models. In mice, even relatively limited binge-like alcohol drinking can lead to disruptions in liver function. Liver CYP2E1 protein levels increased after a single binge-intake session and repeated, excessive alcohol intake by 27% and 57%, respectively. Repeated binge-like intake also increased triglyceride levels in the liver and plasma and increased lipid droplets in the liver.

Interestingly, alcoholic binges caused more liver injury in obese rats than in lean rats, as shown by the increase in ALT activity and by the liver histology and oxidative stress.

Alcohol consumption and obesity produce CYP2E1 induction and anoxia and generate a nitro-oxidative stress and energy depletion in the pericentral zone, where cells are sensitive to hypoxia and where sevoflurane undergoes a CYP2E1-catalyzed biotransformation.

Finally, a single intravenous dose of cefazolin has also been exceptionally reported to induce acute liver injury [15]. The latency period is typically 1 to 3 weeks after exposure. The severity of liver injury is usually mild-to-moderate, and no case of acute liver failure has been reported. Some of the patients with suspected cefazolin toxicity had also received halogenated anesthetics. The pattern of liver injury after cefazolin exposure is assumed to be cholestatic, contrasting with the hepatocellular pattern of the halogenated anesthetics.

In conclusion, after a reasonable exclusion of all other possible causes, sevoflurane anesthesia caused fulminant hepatic failure in a young man who presented with a mild degree of microvesicular steatosis related with obesity
